# Ovarian carcinosarcomas: p53 status defines two distinct patterns of oncogenesis and outcomes

**DOI:** 10.3389/fonc.2024.1408196

**Published:** 2024-08-16

**Authors:** Gurdial Dhillon, Marta Llaurado-Fernandez, Basile Tessier-Cloutier, Keiyan Sy, Dina Bassiouny, Guangming Han, Nelson K. Y. Wong, Kathryn McRae, Mary Kinloch, Jennifer Pors, Laura Hopkins, Allan Covens, Martin Köbel, Cheng-Han Lee, Mark S. Carey

**Affiliations:** ^1^ Department of Obstetrics & Gynaecology, University of British Columbia, Vancouver, BC, Canada; ^2^ Department of Pathology, Faculty of Medicine and Health Sciences, McGill University, Montreal, QC, Canada; ^3^ Department of Pathology, University of Toronto, Toronto, ON, Canada; ^4^ Department of Pathology, Sunnybrook Health Science Centre, Toronto, ON, Canada; ^5^ Department of Pathology and Laboratory Medicine, Surrey Memorial Hospital, Surrey, BC, Canada; ^6^ Department of Experimental Therapeutics, BC Cancer, Vancouver, BC, Canada; ^7^ Department of Pathology and Laboratory Medicine, University of Saskatchewan, Saskatoon, SK, Canada; ^8^ Department of Pathology and Laboratory Medicine, University of British Columbia, Vancouver, BC, Canada; ^9^ Division of Gynaecologic Oncology, Saskatoon Cancer Centre, Saskatoon, SK, Canada; ^10^ Division of Gynaecologic Oncology, Sunnybrook Health Science Centre, University of Toronto, Toronto, ON, Canada; ^11^ Department of Pathology and Laboratory Medicine, University of Calgary, Calgary, AB, Canada; ^12^ Department of Pathology and Laboratory Medicine, University of University of Alberta, Edmonton, AB, Canada; ^13^ Department of Clinical Research, BC Cancer, Vancouver, BC, Canada

**Keywords:** ovarian cancer, ovarian carcinosarcoma, MMMT, immunohistochemistry, p53 IHC, *BRCA*, PARPi

## Abstract

**Objectives:**

Ovarian carcinosarcoma (OCS) is a rare and lethal type of ovarian cancer. Despite its incredibly poor prognosis, it has received little research attention. In this study, we aim to evaluate the molecular features of OCS and elucidate their clinical significance.

**Study methods:**

We examined 30 OCS by immunohistochemistry (IHC) and targeted panel sequencing collected from a single institution (2003–2013) as the initial molecularly characterized cohort (Cohort A). From November 2016 to April 2023, we collected an additional 67 OCS cases from three institutions across British Columbia and Alberta as the contemporary cohort (Cohort B) for clinical correlation. The Kaplan–Meier method was used to estimate overall and progression-free survival, and differences in survival rates were compared using the log-rank test. All tests were two-sided. A *p*-value of less than 0.05 was considered statistically significant.

**Results:**

The majority of OCS (82%) in the initial Cohort A were p53-mutated, and the carcinomatous component displayed the histological and molecular features of a high-grade tubo-ovarian serous carcinoma (HGSC-like). In a minority of OCS, the epithelial components were characteristics of endometrioid or clear cell carcinomas, and IHC staining was wild type for p53. In the contemporary Cohort B, we observed the same histological findings related to the p53 IHC staining pattern. The median overall survival of the p53-mutated HGSC-like OCS (47 patients) was significantly higher (43.5 months) compared with that of the p53 wild-type OCS (10 patients, 8.8 months; *P* < 0.01). Pathogenic *BRCA1/2* germline/somatic mutations were observed in 7 patients (17.5%) of HGSC-like OCS, and all these patients were alive at 3 years from diagnosis compared to a 51% 3-year survival among the patients with *BRCA1/2* wild-type HGSC-like OCS (33 patients) (*p* = 0.022). Majority of patients (6/7) with *BRCA1/2*-mutated OCS received poly (ADP-ribose) polymerase inhibitor as maintenance therapy in this cohort.

**Conclusions:**

Most OCSs have a morphologic and molecular profile resembling HGSC; however, some OCSs display a molecular profile that suggests origin through non-serous oncogenic pathways. This molecular distinction has both prognostic and treatment (predictive) implications. These findings underscore the importance of routine p53 IHC testing on all OCS and *BRCA1/2* testing on p53-mutated OCS.

## Introduction

Ovarian carcinosarcoma (OCS) is a rare ovarian malignancy comprising only 1%–4% of all ovarian cancers (1–4). For some time, it was thought that OCS was a distinct sarcoma type within ovarian malignancies, unrelated to the more common epithelial ovarian cancers. We now appreciate that gynecologic carcinosarcomas (CSs) represent an epithelial metaplastic carcinoma with sarcomatous transdifferentiation. This was originally demonstrated using ultrastructural and immunohistochemical studies on these cancers; however, subsequent genomic analyses have revealed that the carcinomatous and sarcomatous components are clonally related and their mutational profiles more closely resemble the usual types of epithelial carcinomas arising from the ovary (5–12). Applying The Cancer Genome Atlas (TCGA) endometrial cancer molecular classification, Gotoh et al. recently examined 109 gynecologic CSs that included 17 OCSs and found that the majority (88%) exhibited a copy number–high molecular profile that was enriched by the presence of *TP53* mutation, whereas the rest exhibited a copy number–low molecular profile (13). None of the OCSs examined were *POLE* ultramutated or microsatellite unstable. These findings suggest underlying heterogeneity in the oncogenesis of OCS. More recent studies in OCS have also shown that approximately 80% were *TP53*-mutated and displayed WT-1 expression, which is characteristic for high-grade tubo-ovarian serous carcinomas (HGSCs) (4, 9, 13, 14). Some also occur in the presence of serous tubal intraepithelial carcinoma lesions or recur as CS after initially presenting as HGSC (15–17). Despite these molecular similarities, OCSs often have a more aggressive clinical course, with a significantly worse 5-year survival compared to HGSC (2–4, 18). This survival difference may be related to adverse prognostic factors such as advanced stage, suboptimal surgical cytoreduction, presence of heterologous sarcomatoid features on histopathology, increased expression of vascular endothelial growth factor, as well as differences in treatment response as OCSs typically respond poorly to platinum and taxane-based chemotherapy (19–22). Furthermore, poly (ADP-ribose) polymerase inhibitors (PARPis) are now routinely used for treating homologous recombination–deficient (HRD) HGSC (23, 24). This may also contribute to the discrepant outcomes between HGSC and OCS recognizing that PARPi may not be used to treat OCS. We know that, within HGSC, *BRCA1/2* mutation status remains a significant prognostic biomarker for overall survival (OS) (25). There are few reports characterizing *BRCA1/2* mutation status in OCS although a recent study demonstrated pathogenic *BRCA1/2* mutations in 5 of the 49 (10.2%) patients (26). Moreover, aside from a few case reports, PARPi response data and patient outcomes information in *BRCA1/2*-mutated OCS are lacking.

The goal of our study was to examine a series of OCS and evaluate the prognostic and therapeutic significance of p53 immunohistochemistry (IHC) and *BRCA1/2* status in OCS.

## Methods

### Cohort A: initial cohort for molecular characterization

#### Study samples

We examined an initial cohort of 30 OCS cases collected from 2003 to 2013 at Sunnybrook Hospital (Toronto, Canada). Each case was reviewed to confirm the diagnosis by an expert gynecologic pathologist. A tissue microarray was constructed with duplicate 1-mm tissue cores from the carcinomatous and sarcomatous components, respectively, for each of the 30 cases. Ethical approval for the study was obtained from the institutional research board.

#### Immunohistochemistry

IHC was performed on the tissue microarray. The primary antibodies used were as follows: Paired Box 8 (PAX 8) (clone BC12/ACI 438, 1:100, Biocare Medical Concord, California, USA), Wilms Tumor 1 (WT1) (clone 6F-H2, ready-to-use, Dako, Burlington, Ontario, Canada), Estrogen Receptor (ER) (clone SP1, RM-9101, 1:25, Thermo Fisher Scientific, Ottawa, Ontario, Canada), Tumor protein P53 (p53) (clone DO-7, 1:800, M7001, Dako, Burlington, Ontario, Canada), DNA mismatch repair protein Mlh1 (MLH 1) (clone ES05, 1:100, Dako, Burlington, Ontario, Canada), DNA mismatch repair protein Msh2 (MSH2) (clone 25D12, prediluted, NCL), MSH6 (clone 44/MSH6, 1:2000, BD Biosciences), and DNA mismatch repair endonuclease postmeiotic segregation increased 2 (PMS2) (clone A16-4, 1:100, BD Biosciences). The unstained slides were processed using the Ventana Discovery XT and the Ventana Benchmark XT automated system (Ventana Medical Systems, Tucson, Arizona, USA) as per the manufacturer’s protocol with proprietary reagents. Heat-induced antigen retrieval method was used in the Cell Conditioning Solution (CC1-Tris–based EDTA buffer, pH 8.0, Ventana). The Ventana Universal Secondary Antibody was used for 32 min at 37°C. The detection system used was the Ventana DABMap kit and the Ventana OptiView DAB kit.

For PAX8, ER, WT1, MLH1, PMS2, MSH2, and MSH6, only nuclear staining was considered and evaluated; the carcinomatous and sarcomatous components were evaluated separately. PAX8 and ER immunostains were scored as positive if greater than 10% of the cells exhibited moderate to strong positive (definite) nuclear staining. p53 expression was interpreted in both the carcinomatous and sarcomatous components using established published criteria (27). Staining was considered to be mutation-type/aberrant/abnormal if the tumor showed: (i) diffuse moderate to strong uniform nuclear staining in ≥80% of the tumor cells (p53 overexpression mutation pattern); (ii) diffuse complete absence of nuclear staining in the tumor cells in the presence of focal nuclear staining of the stromal cells as an internal positive control (p53 absent expression mutation pattern); or (iii) diffuse cytoplasmic staining (p53 cytoplasmic mutation pattern). p53 expression was classified as wild type in cases with nuclear staining involving <80% of the tumor cells, displaying variable intensity.

#### DNA extraction and targeted sequencing

For each case, paraffin scrolls (3 µm × 20 µm) from a tumor-rich tumor block (greater than 50% tumor content) containing both the carcinomatous and sarcomatous components were obtained. DNA was extracted from the paraffin scrolls using the Qiagen formalin-fixed paraffin-embedded tissue DNA extraction kit based on the manufacturer’s protocols. We performed sequencing analysis to detect mutations in 26 genes that have been previously found to be recurrently mutated in carcinomas of the gynecologic tract as described previously (28). These included the full coding regions of *AKT1*, *ARID1A*, *FBXW7*, *FGFR2*, *JAK1*, *KRAS*, *MLH1*, *MSH2*, *MSH6*, *NRAS*, *PIK3CA*, *PIK3R1*, *PIK3R2*, *PMS2*, *POLE*, *PPP2R1A*, *PTEN*, *RNF43*, *RPL22*, *SMARCA4*, *STK11*, *SPOP*, and *TP53* in selected exon in *CTNNB1* (exon 3). The Illumina custom TruSeq amplicon panel was designed using Illumina’s DesignStudio and included 1,173 amplicons (175 bp) that covers 98% of the exons and untranslated regions of these 26 genes. Custom amplicon libraries were prepared starting with 250 ng of DNA as per the Ilumina’s Custom TruSeq Library Preparation protocol. Before pooling, normalization was performed by quantifying individual libraries using the Qubit fluorometer and then pooled on the basis of equal concentrations. Library pools were then quantitated for amplifiable libraries using the Kapa Biosystems FAST qPCR SYBR quantification kit on the basis of the manufacturer’s protocols. Pooled TruSeq libraries were sequenced using the Illumina MiSeq using 300 cycle V2 kits. Analysis was performed using the MiSeq Reporter and somatic variant caller 3.2.3.0. Only non-synonymous mutations passing quality filter with at least 10% variant allele frequency were further evaluated. These mutations were manually checked in bam files using Integrated Genome Viewer.

### Cohort B: contemporary cohort for clinical correlation

We then collected contemporary OCS cases from three institutions [BC Cancer Agency (Vancouver, BC, Canada), University of Alberta Cancer Center (Edmonton, AB, Canada), and University of Calgary (Calgary, AB, Canada)] from November 2016 to April 2023. This population-based contemporary cohort was assembled to address questions related to tumor type, p53 status, *BRCA1/2* mutation status, and clinical outcome in the PARPi era. Each case was reviewed by a subspecialty pathologist in gynecologic pathology who verified the presence of the carcinomatous and sarcomatous components. *BRCA1/2* mutation status (if performed as part of the routine clinical care), treatment, and clinical outcome data were collected. The study was approved by institutional research boards. Participant consent was waived because of the minimal risk and the retrospective nature of the study. OS was calculated as the time from the date of pathologically confirmed diagnosis till death or date of last known follow-up. Progression-free survival (PFS) was reported as the time from date of diagnosis to the time of progression, recurrence, or death. Majority of patients (6/7) with *BRCA1/2*-mutated OCS received PARPi as part of their therapy.

### Statistical analysis

Demographics and baseline characteristics were summarized using descriptive statistics (N, median, and range) for continuous variables and N (%) for discrete variables. The Student’s t-test was used to compare means between two groups. The Kaplan–Meier method was used to estimate the OS, and the stratified log-rank test was used to assess survival differences. All tests were two-sided. A *p-*value of less than 0.05 was considered statistically significant.

## Results

### Molecular analysis of study Cohort A demonstrates heterogeneity in OCS

The results of the molecular analysis (DNA sequencing panel and IHC panel) are summarized in **Table 1**, and additional IHC results and clinical information are shown in **Supplementary Table 1**. Of the 30 OCSs studied, 26 (86.7%) demonstrated genetic and immunohistochemical (IHC) evidence of a *TP53* mutation. There were 24 tumors that harbored *TP53* mutations, 15 tumors that harbored missense mutations, five tumors that harbored frame-shift mutations, three tumors with non-sense mutations, and one tumor that had both a non-sense and a frameshift mutation. By p53 IHC, 26 tumors exhibited mutation staining patterns, with 16 tumors showing overexpression mutation pattern and 10 tumors showing absent expression mutation pattern. Of note, all eight tumors harboring either a missense or a frameshift *TP53* mutation exhibited absent expression mutation-pattern p53 staining, which suggests that there was likely concurrent loss of heterozygosity in *TP53*. The single tumor that harbored both a frameshift and a nonsense (R210X) *TP53* mutations exhibited diffuse expression p53 mutation pattern. There were two OCSs without demonstrable single-nucleotide variation or small insertion/deletion (indel) by targeted sequencing and both exhibited absent expression mutation pattern by p53 IHC. In all cases with mutation-pattern p53 staining, the carcinomatous and sarcomatous components showed concordant p53 staining result and pattern. All 26 OCSs that demonstrated genetic and/or immunohistochemical evidence of TP53 mutation were DNA mismatch repair (MMR)-intact, with 20 tumors (77%) exhibiting WT1 nuclear expression and 17 tumors (65%) exhibiting ER expression in the carcinomatous component by IHC. Four of the 30 (13%) OCSs lacked evidence of *TP53* mutation by genetic and IHC analyses, and three of the four tumors harbored mutations involving *KRAS* (one G12A and one G12D), *RPL22* (one frameshift), *ARID1A* (one frameshift), and/or *CTNNB1* (one with S37C) that are often seen in non-HGSC ovarian carcinomas. These four tumors also lacked WT1 expression and were MMR-intact; two of the four tumors were ER-positive. In terms of PIK3CA pathway alterations, five tumors harbored exon 9 or 20 hotspot activating *PIK3CA* mutations (including three of the four TP53 wild-type CSs). None of the OCS examined harbored pathogenic *POLE* exonuclease domain mutations, although one tumor was found to have a non-sense mutation (Q1625X) outside of exonuclease domain. None of the tumors showed human epidermal growth factor receptor 2 (HER2) overexpression by IHC and the sarcomatous component in all 30 CSs consistently lacked nuclear expression of PAX8, ER, and WT1, including cases where the corresponding carcinomatous component showed expression for these proteins. All tumors showed intact expression of ARID1A except for the one tumor with wild-type *TP53.* This cancer had a frameshift *ARID1A* mutation. Overall, the findings from Cohort A confirms the molecular heterogeneity of OCS, with the majority showing a HGSC-like p53-mutated profile in the carcinomatous component and a minority (cases 6, 10, 25, and 28) showing a p53 wild-type non-HGSC profile in the carcinomatous component.

**Table 1:** Summary of immunohistochemistry and targeted sequencing results of 30 ovarian carcinosarcomas (OCS) cases in Cohort A.

Case	TP53 mutation	P53 IHC (CA)	P53 IHC (SA)	Other mutations	MMR	PAX8 (CA)	PAX8 (SA)	WT1 (CA)	WT1 (SA)
**1 **	R141H	Mutated (OE)	Mutated (OE)	*PIK3CA* (*Y644C*)	Normal	Pos	Neg	Pos	Neg
**2 **	R43H	Mutated (OE)	Mutated (OE)		Normal	Pos	Neg	Pos	Neg
**3 **	f.s.	Mutated (AE)	Mutated (AE)	*FBXW7* (*RS9Q*), *PIK3R2* (*R101H*)	Normal	Neg	Neg	Pos	Neg
**4 **	f.s.	Mutated (AE)	Mutated (AE)		Normal	Pos	Neg	Pos	Neg
**5 **	G134R	Mutated (OE)	Mutated (OE)	*KRAS* (*G12D*), *PIK3CA* (*ES45K*), *FBXW7*	Normal	Pos	Neg	Neg	Neg
**6 **	No SNV/indel	Wild-type	Wild-type	*RPL22* (*f.s.*), *ARID1A* (*f.s.*), *PIK3CA* (*R524K*), *MSH6* (*f.s.*), *POLE* (*Q1625X*)	Normal	N/A	Neg	Neg	Neg
**7 **	R81X	Mutated (AE)	Mutated (AE)	*BRCA1* (*D401V*)	Normal	Pos	Neg	Pos	Neg
**8 **	C124X	Mutated (AE)	Mutated (AE)		Normal	Pos	Neg	Pos	Neg
**9 **	H61R	Mutated (OE)	Mutated (OE)	*FGFR2* (*N615I*)	Normal	N/A	Neg	Neg	Neg
**10 **	No SNV/indel	Wild-type	Wild-type	*AKT* (*E17K*), *PIK3CA* (*RS24K*), *CTNNB1* (*537C*)	Normal	Pos	Neg	Neg	Neg
**11 **	R1 75H	Mutated (OE)	Mutated (OE)	*PIK3R2* (*L127F*)	Normal	Pos	Neg	Pos	Neg
**12 **	f.s.	Mutated (AE)	Mutated (AE)	*POLE* (*f.s.*)	Normal	Pos	Neg	Pos	Neg
**13 **	R1 17T	Mutated (OE)	Mutated (OE)		Normal	Pos	Neg	Pos	Neg
**14 **	f.s. R210X	Mutated (OE)	Mutated (OE)		Normal	Pos	Neg	Pos	Neg
**15 **	R81X	Mutated (AE)	Mutated (AE)	*MSH6* (*N742S*)	Normal	Pos	Neg	Pos	Neg
**16 **	I63T	Mutated (OE)	Mutated (OE)		Normal	Pos	Neg	Pos	Neg
**17 **	No SNV/indel	Mutated (AE)	Mutated (AE)		Normal	Pos	Neg	Pos	Neg
**18 **	I63T	Mutated (OE)	Mutated (OE)		Normal	Neg	Neg	Pos	Neg
**19 **	R4 3H	Mutated (OE)	Mutated (OE)		Normal	Neg	Neg	Neg	Neg
**20 **	G113D	Mutated (OE)	Mutated (OE)		Normal	Pos	Neg	Neg	Neg
**21 **	f.s.	Mutated (AE)	Mutated (AE)		Normal	Pos	Neg	Pos	Neg
**22 **	C44Y	Mutated (OE)	Mutated (OE)	*PIK3CA* (*H1047R*), *MSH2* (*Q374H*)	Normal	Pos	Neg	Pos	Neg
**23 **	R1 75H	Mutated (OE)	Mutated (OE)		Normal	Pos	Neg	Neg	Neg
**24 **	f.s.	Mutated (AE)	Mutated (AE)		Normal	Neg	Neg	Pos	Neg
**25 **	No SNV/indel	Wild-type	Wild-type	*KRAS* (*G12A*), *MSH2* (*L279V*)	Normal	Pos	Neg	Neg	Neg
**26 **	V142G	Mutated (OE)	Mutated (OE)	*SPOP* (*D291G*)	Normal	Pos	Neg	Pos	Neg
**27 **	C1 43Y	Mutated (OE)	Mutated (OE)		Normal	Pos	Neg	Pos	Neg
**28 **	No SNV/indel	Wild-type	Wild-type	*PIK3CA* (*E545G*)	Normal	Pos	Neg	Neg	Neg
**29 **	No SNV/indel	Mutated (AE)	Mutated (AE)	*POLE* (*R47W*)	Normal	Pos	Neg	Pos	Neg
**30 **	V142F	Mutated (OE)	Mutated (OE)		Normal	Pos	Neg	Neg	Neg

CA, carcinoma component; SA, sarcoma component; MMR, mismatch repair protein status by immunohistochemistry; OE, overexpression of p53 (mutation pattern); AE, absent expression of p53 (mutation pattern); f.s., frameshift mutation; SNV, single-nucleotide variation; Indel, small insertion or deletion.

### High-grade serous-like OCSs in Cohort B harbor high rates of mutations in high-penetrance homologous recombination–deficient genes, including *BRCA1/2*

**Figure 1:**
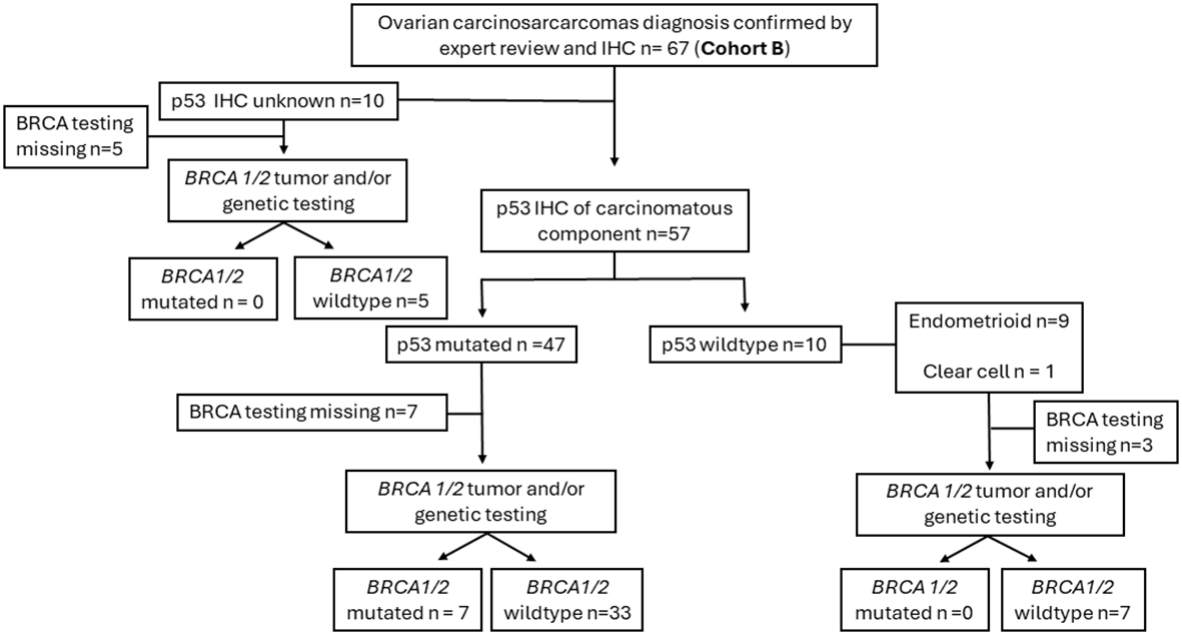
Diagrammatic overview of the ovarian carcinosarcoma in contemporary Cohort B. IHC, immunohistochemistry; BRCA, *BRCA1* and *BRCA2*.

The clinical and molecular features of study Cohort B (67 patients) are summarized in **Table 2**. P53 IHC was performed as part of the pathology diagnostic work-up in 57 of the 67 patients (85%) (**Figure 1**). The great majority (82.5%, 47 of 57) were p53-mutated with a carcinomatous component that displayed histologic features of HGSC. Ten cases showed wild-type p53 expression, and the carcinomatous component in nine of these 10 cases displayed endometroid-type histologic features, with one showing mismatch repair–deficient immunostaining pattern from a patient with known Lynch syndrome. Seven of the 10 wild-type p53 cases had *BRCA1/2* germline/or somatic testing, and none showed and pathogenic *BRCA1/2* mutations. These findings are in keeping with the observation made in Cohort A that the majority of OCSs belong to a HGSC-like group (p53-mutated) with a minority in the non–HGSC-like group characterized by wild-type p53.

**Table 2:** Baseline patient characteristics of Cohort B.

OCS cases		Total	BRCA wild type	BRCA mutated	p53 mutated	p53 wild type
Number of cases		67	45	7	47	10
Age (years)						
	Median Range	67 43–88	65 43–88	69 56–81	68* 43–88	52* 44–76
Stage						
	I II III IV	8 (12) 13 (19) 34 (51) 12 (18)	3 (6) 9 (20) 26 (58) 7 (16)	1 (14) 1 (14) 4 (57) 1 (14)	4 (9) 7 (15) 26 (55) 10 (21)	2 (20) 4 (40) 3 (30) 1 (10)
Neoadjuvant treatment						
	Yes No	15 (22) 52 (78)	14 (31) 31 (69)	0 (0) 7 (100)	13 (28) 34 (72)	1 (10) 9 (90)
First-line treatment						
	Platinum-based chemotherapy PARPi maintenance No systematic therapy	60 (90) 16 (24) 7 (10)	43 (95) 10 (25) 2 (5)	7 (100) 6 (86) 0 (0)	45 (96) 14 (30) 2 (4)	9 (90) 1 (10) 1 (10)
Residual disease						
	Microscopic or less than 1 cm Greater than 1 cm No surgery	41 (61) 25 (37) 1 (2)	27 (60) 18 (40) 0 (0)	6 (86) 1 (14) 0 (0)	29 (62) 18 (38) 0 (0)	5 (50) 4 (40) 1 (10)
Disease status at last follow-up						
	No evidence of disease Alive with disease Died of disease or other cause	16 (24) 9 (13) 42 (63)	9 (20) 5 (11) 31 (69)	2 (29) 4 (57) 1 (14)	12 (26) 9 (19) 26 (55)	1 (10) 0 (0) 9 (90)

OCS, ovarian carcinosarcoma; PARPi, poly (ADP-ribose) polymerase inhibitor.

*There is a statistically significant difference in mean age at diagnosis between the p53 mutated and the p53 wild-type OCS.

We then further examined the 47 HGSC-like OCSs to see if they had tumor or germline *BRCA1/2* testing performed. Among the 40 cases with BReast CAncer gene 1 and 2 (BRCA 1/2) testing, seven (17.5%) harbored pathogenic *BRCA1/2* mutation (three cases germline). Additionally, within the remaining HGSC-like OCS, two patients with wild-type germline *BRCA1/2* carried germline moderate penetrance pathogenic mutation in other HRD genes: one with *RAD51C* c.404G>C mutation and the other with *BRIP1* c.1018C>T mutation. All patients harboring pathogenic germline HRD gene mutation had been referred to hereditary medicine for further counseling. For the 10 non–HGSC-like OCS (wild-type p53), seven had tumor and/or germline *BRCA1/2* testing with no pathogenic mutations involving *BRCA1/2* or other HRD genes such as *PALB2*, *RAD51D/C*, or *BRIP1* identified.

### P53 status and *BRCA1/2* mutation status confer prognostic significance

**Figure 2:**
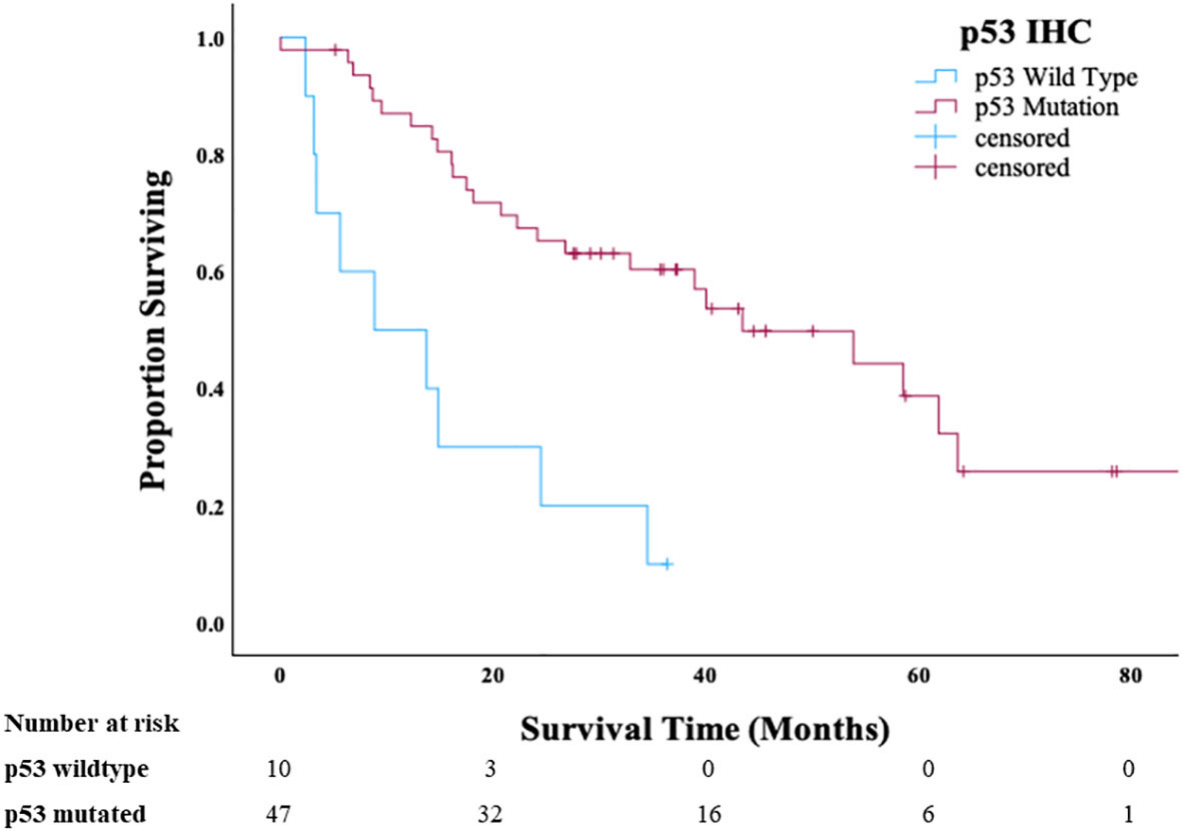
Overall survival of patients with ovarian carcinosarcoma in Cohort B stratified by p53 status.

We subsequently evaluated the clinical outcome of the contemporary Cohort B in relation to tumor molecular groups (p53-mutated HGSC-like or p53 wild-type non–HGSC-like). Patients with p53 wild-type (non–HGSC-like) OCS had significantly shorter median OS (8.8 months) compared with patients with p53-mutated HGSC-like OCS (43.5 months) (*P <* 0.01) (**Figure 2**). There was also a statistical difference in PFS between p53 wild-type (non–HGSC-like) and p53-mutated HGSC-like OCS (P < 0.001) (**Supplementary Figure 1**). There were no apparent confounding clinical features that accounted for the observed difference in survival between the p53 mutant versus the p53 wild-type OCS. Patients were younger at diagnosis in the p53 wild-type group (*p* = 0.02); however, there were no significant differences in stage, use of neoadjuvant chemotherapy, or residual disease between the two groups (**Table 2**).

**Figure 3:**
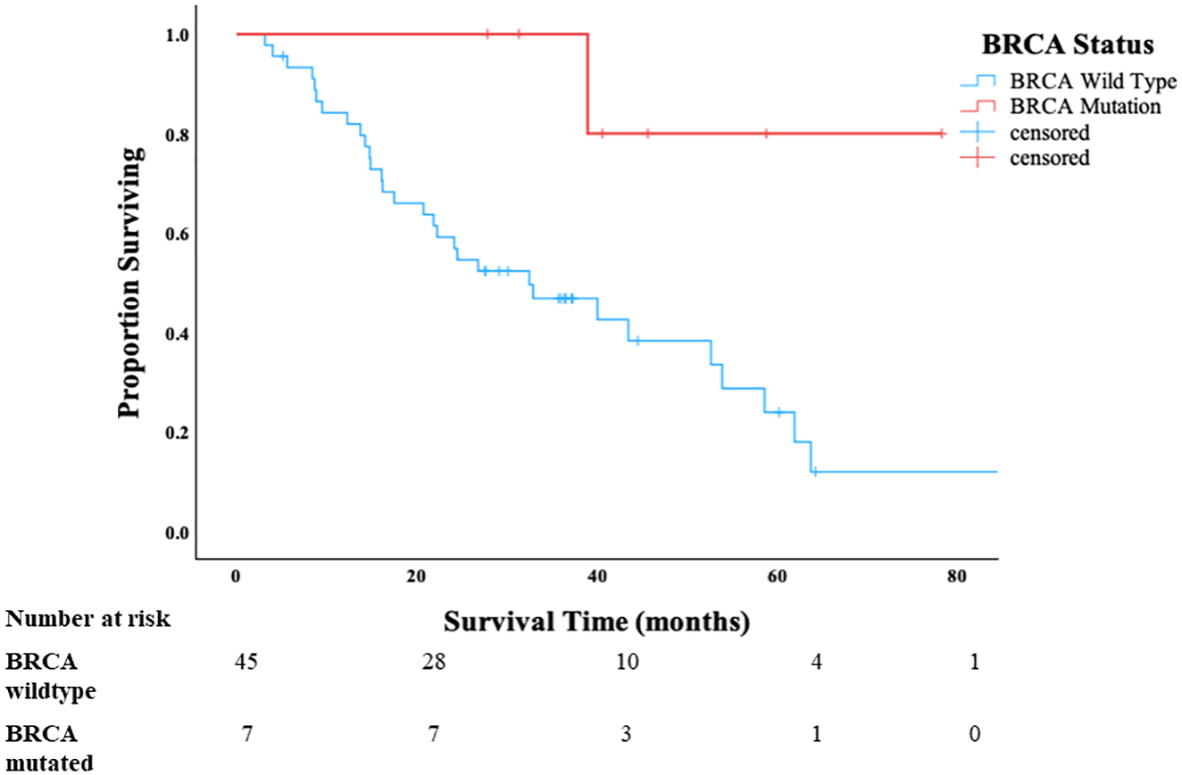
Overall survival of patients with p53-mutated HGSC-like ovarian carcinosarcoma in Cohort B stratified by *BRCA1/2* status.

In the p53-mutated HGSC-like OCS cases, all patients with *BRCA1/2* mutation were alive at 3 years compared to 51% of patients with wild-type *BRCA1/2* status (*p* = 0.022) (**Figure 3**). Once again, there were no apparent differences in clinical factors (age, stage, use of neoadjuvant chemotherapy, and residual disease), between the BRCA1/2-mutated and BRCA1/2 wild-type groups that would account for the observed difference in survival (**Table 2**). As expected, PFS was longer in the BRCA-mutant cases compared to wild-type p53; however, this difference did not reach statistical significance (*p* = 0.12) (**Supplementary Figure 2**).

## Discussion

OCS is an uncommon but highly aggressive histotype of ovarian carcinoma and is believed to arise through sarcomatous transformation (epithelial mesenchymal transition) of the epithelium. Its uncommon nature has limited our understanding of this cancer. The primary treatment strategy for OCS remains a combination of primary cytoreductive surgery and platinum-based chemotherapy, with emerging potential seen with immunotherapy and targeted therapies (29). The utilization of comprehensive molecular testing could improve outcomes by facilitating tailored treatments for particular patient cohorts. Here, we molecularly characterized a series of OCS and confirm the presence of molecular heterogeneity within OCS. We have shown that the majority of OCSs examined have mutation and immunophenotypic features that resemble high-grade serous carcinomas of tubo-ovarian origin (HGSC-like OCS). This is in keeping with the notion of OCS representing a type of metaplastic carcinoma and suggests that many have evolved through a HGSC oncogenic pathway. Conversely, a small subset of OCS exhibits a mutation and immunophenotypic profile that are not compatible with an origin from HGSC (non–HGSC-like OCS). The profiles in these cases more closely resemble ovarian endometrioid or clear cell-type carcinoma, and all are p53 wild type. This suggests that that a minor subset of OCS can arise through endometrioid/clear cell carcinoma oncogenic pathways as previously suggested (30–35). Our findings challenge the notion that all OCSs are variant of HGSC but perhaps represent a distinct metaplastic subtype that likely evolved through serous type or non-serous type oncogenic pathways.

When looking at the clinical outcomes of Cohort B, we found that separating OCS into HGSC-like and non–HGSC-like groups based on TP53 status have clinical implications with regard to survival. Here, we observed that HGSC-like OCS (p53-mutated) and non–HGSC-like OCS (p53 wild type) have different survival outcomes. Although histologic subtyping of the carcinomatous component alone has not been associated with differential survival outcomes in the past, it is worth noting that the use of TP53 IHC provides a more objective and accurate method of subtyping OCS into a HGSC-like and non–HGSC-like groups. Furthermore, the difference in survival observed in this contemporary cohort may also be partially attributed to access to PARPi that may have increased survival in this group. PARPis have changed the treatment paradigm for ovarian cancer patients and have remarkable efficacy, particularly in HRD ovarian carcinomas. Based on our results, we advocate for the routine use of TP53 IHC analysis to subtype OCS into HGSC-like and non–HGSC-like groups. Furthermore, all HGSC-like OCSs should be sent for *BRCA1/2* testing to identify patients eligible for PARPi therapy.

A contemporary review of endometrial CS recently suggested that p53 wild-type CS may, in fact, represent misclassified endometrioid carcinomas with reactive stroma or spindle cell growth, and they found that all endometrial CS in their study were p53 abnormal (36). Hence, it is possible that our p53 normal OCS were misclassified ovarian endometrioid or clear cell carcinomas with desmoplastic stroma or spindle cell growth. While there is no objective gold standard, all our cases underwent expert pathology review. Furthermore, the shorter survival of p53 wild-type OCS compared to p53 mutant OCS argues against misclassification because patients with ovarian endometrioid carcinomas have a longer survival compared to HGSC (3). Nevertheless, we support the recommendation that all p53 wild-type gynecologic CSs warrant pathology review to exclude mimics (36).

Another important finding in this study relates to the poor prognosis of patients with p53 wild-type OCS. In both Cohorts A and B, the adenocarcinoma component of these OCSs was usually endometrioid/clear cell histology. These OCSs frequently contain mutations in *KRAS* or *PIK3CA*, resulting in upregulation of their respective pathways. Upregulated phosphatidylinositol 3-kinase (PI3K) pathway can play an important role in chemoresistance and preservation of genomic stability (37). Alternate therapies for these patients represent an urgent unmet need, and novel agents targeting *KRAS* or *PIK3CA* mutations should be evaluated (38).

In the contemporary Cohort B, it should be noted that there was only one MMR-deficient OCS in a patient with a known Lynch Syndrome. Although uncommon, MMR deficiency in OCS may represent another opportunity for tumor-agnostic therapy, as there have been two landmark studies showing a remarkable survival benefit using checkpoint inhibition in MMR-deficient endometrial cancer (39, 40). Therefore, another consideration is to perform MMR IHC or microsatellite instability testing in non–HGSC-like p53 wild-type OCS.

*POLE* exonuclease domain mutations were not identified in the current molecular cohort (Cohort A) of OCS. This is not unexpected as the great majority of OCS appears to arise through HGSC-like pathway in our molecular cohort and pathogenic *POLE* mutations are never seen in serous tubo-ovarian carcinoma. Evidence of *POLE* exonuclease domain mutations in p53 wild-type OCS does not exist outside of the case reports of sarcomatous transformation of *POLE*-mutated endometrioid endometrial carcinomas (41). Because these cases are associated with ultra-mutated profiles and indolent behavior, designating them as CS does not reflect their true biology because *POLE*-mutated endometrioid carcinomas often show areas of low-grade atypia inconsistent with the definition of a CS (42, 43).

### Strengths and limitations

The main strength of our study includes expert pathology review of our OCS cases along with detailed clinical annotation and outcomes data for a contemporary cohort of patients. Our study is limited by a relatively small sample size (for both the molecular analysis Cohort A and contemporary Cohort B) limiting the ability to perform multivariable analyses. Thus, our findings require further validation in other contemporary cohorts. The evolving management of OCS, particularly with the advent of PARPi was addressed through the analysis of a contemporary cohort, as the initial molecular cohort analysis predated the clinical use of PARPi.

## Conclusions

Our results show that, based on histological and molecular profiles, OCS can be divided into p53-mutated (HGSC-like) and p53 wild-type (non–HGSC-like) molecular subtypes. Because this molecular distinction suggests different oncogenic pathways and differences in survival and response to therapy, we recommend routine p53 IHC in all OCSs. All p53-mutated cases should be referred for somatic and germline *BRCA1/2* testing due to high percentage (approximately 20%) of these cases harboring pathogenic *BRCA1/2* mutations. P53 wild-type OCSs should be confirmed by gynecological pathology subspecialty review and then undergo MMR IHC and *POLE* genetic testing, if feasible.

## Data Availability

The original contributions presented in the study are included in the article/**Supplementary Material**. Further inquiries can be directed to the corresponding author.
