# Supplementary figures and images for: Ovarian carcinosarcomas: p53 status defines two distinct patterns of oncogenesis and outcomes
Source: Front Oncol. 2024 Aug 16;14:1408196. doi: 10.3389/fonc.2024.1408196 (PMC11361923; doi:10.3389/fonc.2024.1408196)

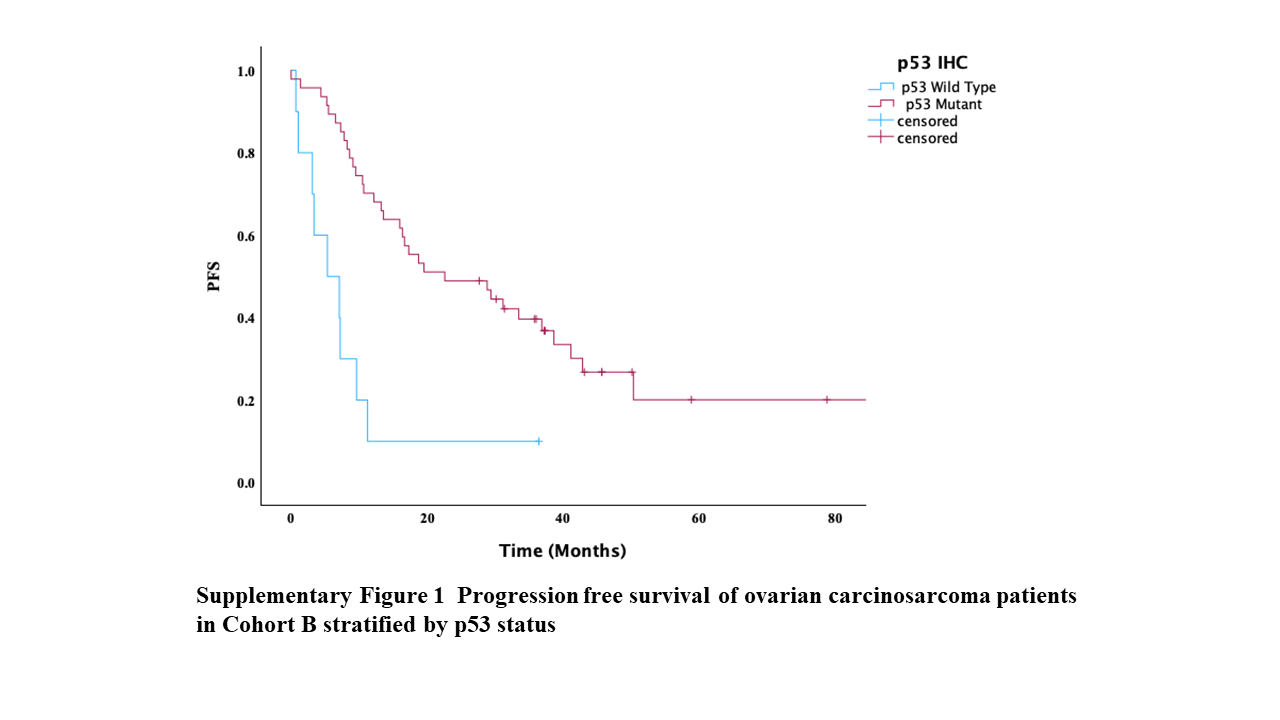

Supplement: Supplementary file 1 [file Image1.tif]

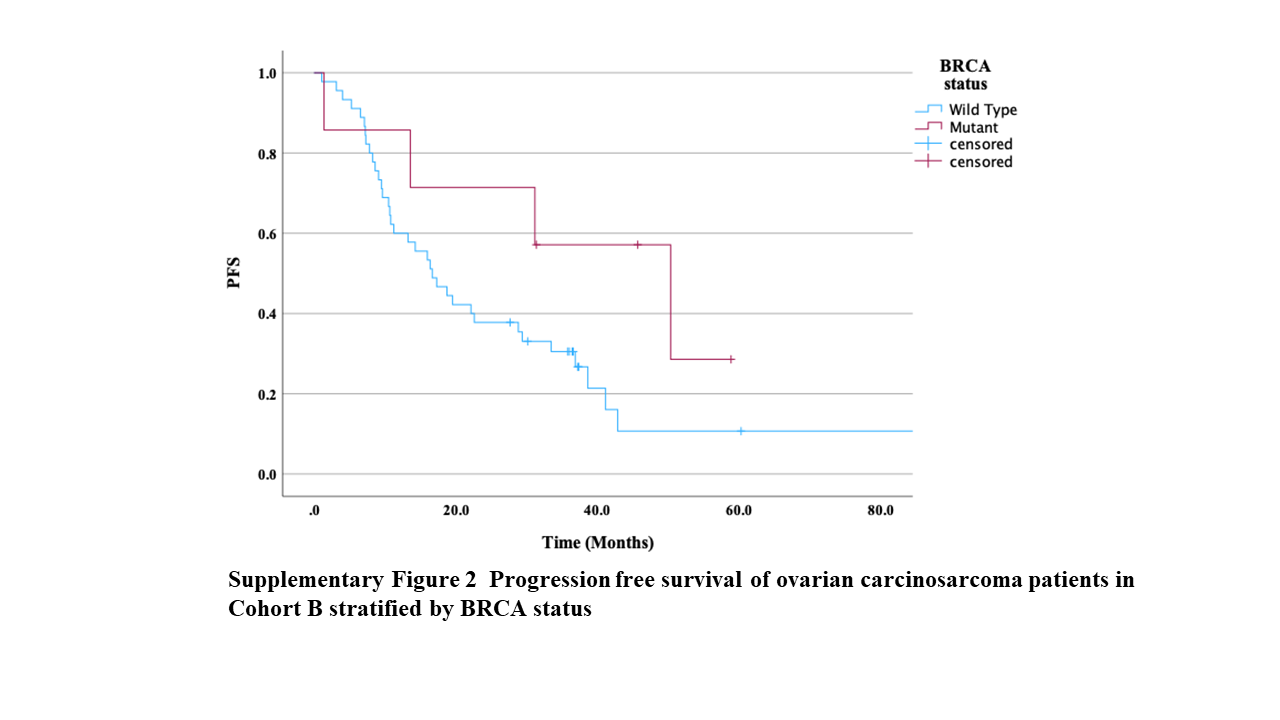

Supplement: Supplementary file 2 [file Image2.tif]
