# Supplementary material for: Ovarian carcinosarcomas: p53 status defines two distinct patterns of oncogenesis and outcomes
Source: Front Oncol. 2024 Aug 16;14:1408196. doi: 10.3389/fonc.2024.1408196 (PMC11361923; doi:10.3389/fonc.2024.1408196)
Supplement: Supplementary file 3 [file Table1.docx]

| **Supplementary Table 1: Clinical and additional immunohistochemical features of cohort A.** pTNM: pathologic TNM staining; DOD: died of disease, DOUC: died of other cause; LFU: lost to follow-up, NA: not available; ER: estrogen receptor, CA: carcinomatous component, SA: sarcomatous component | | | | | | | | | | | |
| --- | --- | --- | --- | --- | --- | --- | --- | --- | --- | --- | --- |
| **Case** | **Age** | **Tumor stage pTNM** | **FIGO Stage** | **Follow up length (months)** | **Status at last follow-up** | **ER-CA** | **ER-SA** | **MLH1** | **MSH2** | **MSH6** | **PMS2** |
| 1 | 63 | T3c Nx Mx | 3 | 19 | DOD | Neg | Neg | Intact | Intact | Intact | Intact |
| 2 | 58 | T3c Nx Mx | 3 | 21 | DOD | Pos | Neg | Intact | Intact | Intact | Intact |
| 3 | 90 | T3b Nx Mx | 3 | 3 | DOD | Neg | Neg | Intact | Intact | Intact | Intact |
| 4 | 70 | T2 Nx Mx | 2 | 53 | DOD | Neg | Neg | Intact | Intact | Intact | Intact |
| 5 | 53 | T3c N0 Mx | 3 | 2 | DOD | Neg | Neg | Intact | Intact | Intact | Intact |
| 6 | 37 | T3c N0 Mx | 3 | 11 | DOD | Neg | Neg | Intact | Intact | Intact | Intact |
| 7 | 63 | T3c Nx Mx | 3 | 13 | DOD | Neg | Neg | Intact | Intact | Intact | Intact |
| 8 | 71 | T3c Nx Mx | 3 | LFU | NA | Pos | Neg | Intact | Intact | Intact | Intact |
| 9 | 57 | T2NxMx | 2 | 14 | DOUC | NA | NA | Intact | Intact | Intact | Intact |
| 10 | 48 | T3c Nx Mx | 3 | LFU | NA | Pos | Neg | Intact | Intact | Intact | Intact |
| 11 | 49 | T3c Nx Mx | 3 | 13 | DOD | Pos | Neg | Intact | Intact | Intact | Intact |
| 12 | 72 | T3c N1 Mx | 3 | 2 | DOD | Pos | Neg | Intact | Intact | Intact | Intact |
| 13 | 52 | T3c N1 Mx | 3 | LFU | NA | Pos | Neg | Intact | Intact | Intact | Intact |
| 14 | 85 | T3c N1 Mx | 3 | LFU | NA | Pos | Neg | Intact | Intact | Intact | Intact |
| 15 | 63 | T3c Nx Mx | 3 | 132 | NED | Pos | Neg | Intact | Intact | Intact | Intact |
| 16 | 69 | T3c Nx Mx | 3 | LFU | NA | Pos | Neg | Intact | Intact | Intact | Intact |
| 17 | 77 | T3c Nx Mx | 3 | LFU | NA | Pos | Neg | Intact | Intact | Intact | Intact |
| 18 | 84 | T3c Nx Mx | 3 | 2 | DOUC | Neg | Neg | Intact | Intact | Intact | Intact |
| 19 | 67 | T3c Nx Mx | 3 | 25 | DOUC | Neg | Neg | Intact | Intact | Intact | Intact |
| 20 | 74 | T3c N1 Mx | 3 | LFU | NA | Pos | Neg | Intact | Intact | Intact | Intact |
| 21 | 54 | T3c Nx Mx | 3 | 11 | DOD | Pos | Neg | Intact | Intact | Intact | Intact |
| 22 | 59 | T3c Nx Mx | 3 | LFU | NA | Pos | Neg | Intact | Intact | Intact | Intact |
| 23 | 69 | T3c Nx Mx | 3 | 18 | DOD | Pos | Neg | Intact | Intact | Intact | Intact |
| 24 | 81 | T3b Nx Mx | 3 | LFU | NA | Pos | Neg | Intact | Intact | Intact | Intact |
| 25 | 62 | T1c Nx Mx | 1 | 57 | DOD | Neg | Neg | Intact | Intact | Intact | Intact |
| 26 | 73 | T3c Nx Mx | 3 | LFU | NA | Pos | Neg | Intact | Intact | Intact | Intact |
| 27 | 66 | T2b Nx Mx | 2 | <1 | DOD | Pos | Neg | Intact | Intact | Intact | Intact |
| 28 | 63 | T3c Nx Mx | 3 | 20 | DOD | Pos | Neg | Intact | Intact | Intact | Intact |
| 29 | 66 | T3c Nx Mx | 3 | 28 | DOD | Pos | Neg | Intact | Intact | Intact | Intact |
| 30 | 64 | T2b Nx Mx | 2 | 19 | DOD | Neg | Neg | Intact | Intact | Intact | Intact |
